# Effective superior vena cava isolation using a novel C-shaped approach

**DOI:** 10.3389/fcvm.2023.1253912

**Published:** 2023-09-15

**Authors:** Chun-Kai Chen, Chih-Chieh Yu

**Affiliations:** ^1^ Division of Cardiology, Department of Internal Medicine, National Taiwan University Hospital Hsin-Chu Branch, Hsinchu, Taiwan; ^2^ Division of Cardiology, Department of Internal Medicine, National Taiwan University Hospital, Taipei, Taiwan; ^3^ Department of Internal Medicine, College of Medicine, National Taiwan University, Taipei, Taiwan

**Keywords:** atrial fibrillation, superior vena cava, isolation, non-circumferential ablation, novel approach

## Abstract

**Introduction:**

Superior vena cava (SVC) isolation has been proposed as part of the ablation strategy for atrial fibrillation. However, circumferential isolation of the SVC can lead to late-onset complications, such as SVC stenosis.

**Methods:**

We describe a detailed observation of the SVC conduction pattern and present a newly developed approach for SVC isolation that involves creating a C-shaped non-circumferential ablation line while sparing the lateral segment.

**Results:**

Twelve consecutive patients were included in the study, all of whom achieved bidirectional block during the ablation procedure.

**Discussion:**

This approach to SVC isolation is effective and has the potential to reduce ablation related complications; however, larger studies and long-term follow-up is warranted to confirm these findings.

## 1. Introduction

Atrial fibrillation (AF) is a common cardiac arrhythmia affecting millions of individuals worldwide. Catheter ablation has become a cornerstone of AF management, with the primary goal of achieving electrical isolation of the pulmonary veins (PVs) (1). Late-onset complications, such as PV stenosis, are associated with the application of ablation energy inside the PVs; the risks of these complications have been significantly reduced by relocating the ablation lines to the antral area. The superior vena cava (SVC) has been reported as a potential source of non-PV triggers of AF, and SVC muscle sleeve length has been linked to AF recurrence after PV isolation (2). However, SVC isolation has been associated with acute phrenic nerve injury and late SVC stenosis. Segmental catheter ablation has been proposed as a safe and effective alternative for achieving complete SVC isolation (3). However, the heterogeneous distribution of muscle sleeves among patients makes this process challenging.

In this study, we introduced a novel approach for SVC isolation using a C-shaped non-circumferential ablation line that spares the lateral segment. This approach minimizes the risk of sinus node injury and latent SVC strictures, and potentially reduces the risk of phrenic nerve injury. Furthermore, this technique can be easily applied to all patients who require SVC isolation, making it a valuable addition to current ablation procedures.

## 2. Methods

The authors confirm that written consent for the submission and publication of this study was obtained from the patients, in line with the COPE guidelines.

Following a clinical diagnosis of AF, the patients underwent ablation following discussions with their physicians. The procedures were performed under propofol sedation and vascular tunnels were accessed under echocardiographic guidance. A transeptal puncture was performed under fluoroscopic guidance. The procedures were performed using the CARTO 3 mapping system with a 20-pole circular mapping catheter and a contact-force irrigation ablation catheter (THERMOCOOL SMARTTOUCH SF Catheter; Biosense Webster, Irvine, CA, USA), in a power-controlled mode (40 W) and using the ablation index module (400 in the posterior/inferior and 450–500 in the anterior/superior segments). An isoproterenol inducibility test was performed (up to 20 mcg/min) to detect PV isorhythms and uncover potential non-PV triggers. If an SVC trigger was identified or if delayed SVC activation was observed during sinus rhythm, SVC isolation was performed.

**Figure 1:**
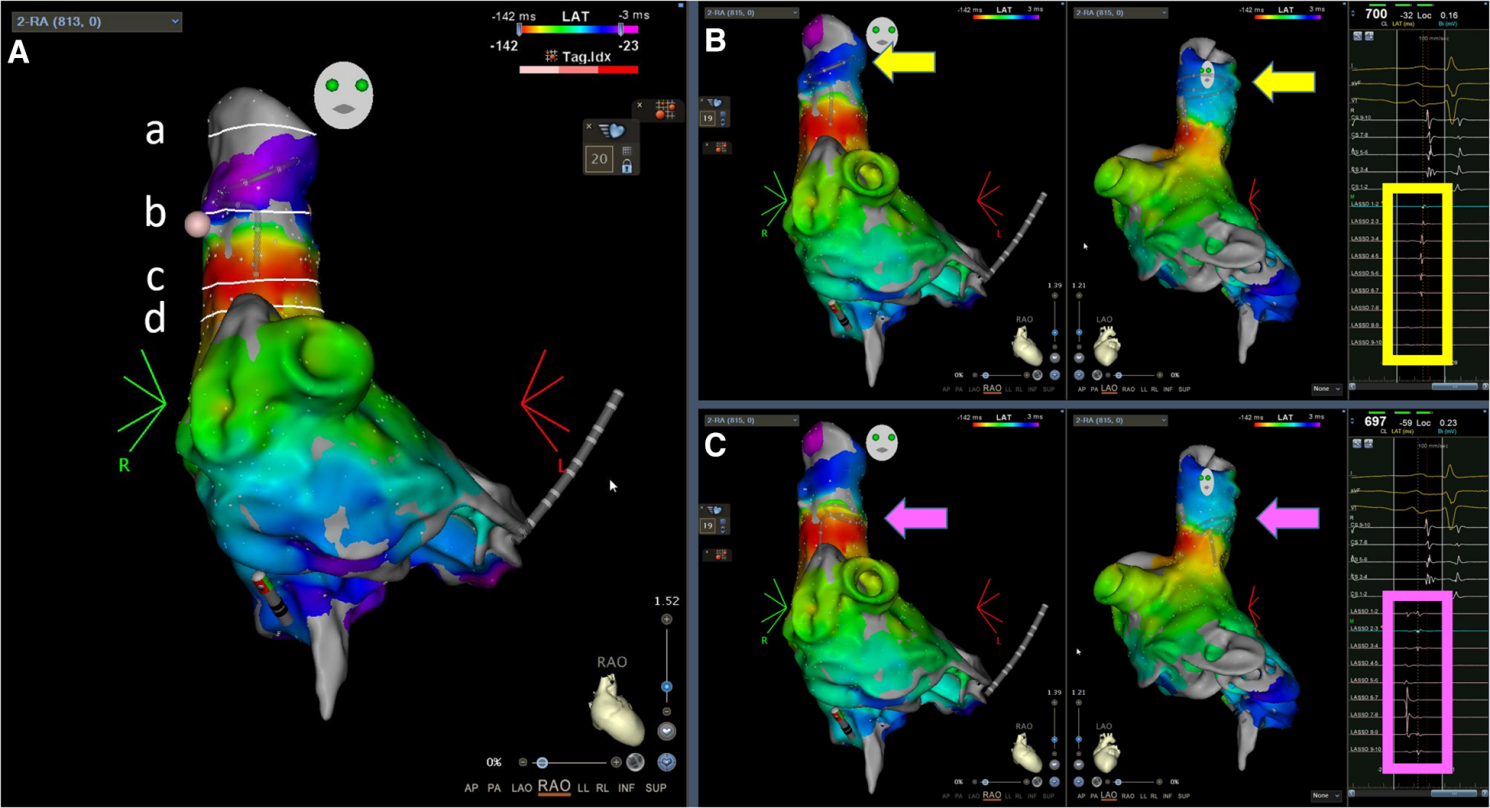
Demonstration of the landmarks and the representative local electrograms. (**A**) Landmarks, from top to bottom: (a) end of the muscle sleeves, (b) ablation line, (c) earliest activation site during sinus rhythm, and (d) anatomical SVC—RA junction in the RAO 30° view; (**B**) Activation maps during sinus rhythm, RAO and left anterior oblique 60° views; the Lasso electrograms demonstrate clear SVC signals without far-field RA signals; (**C**) The same activation maps during sinus rhythm as in (**B**), but the Lasso catheter was placed lower, at the electrical SVC-RA junction where sharp SVC potentials were superimposed with blunt far-field RA electrograms. The arrows indicate the locations of the Lasso catheter. The square frames highlight the electrograms at the location of the Lasso catheter. SVC, superior vena cava; RA, right atrium; RAO, right anterior oblique.

**Figure 2:**
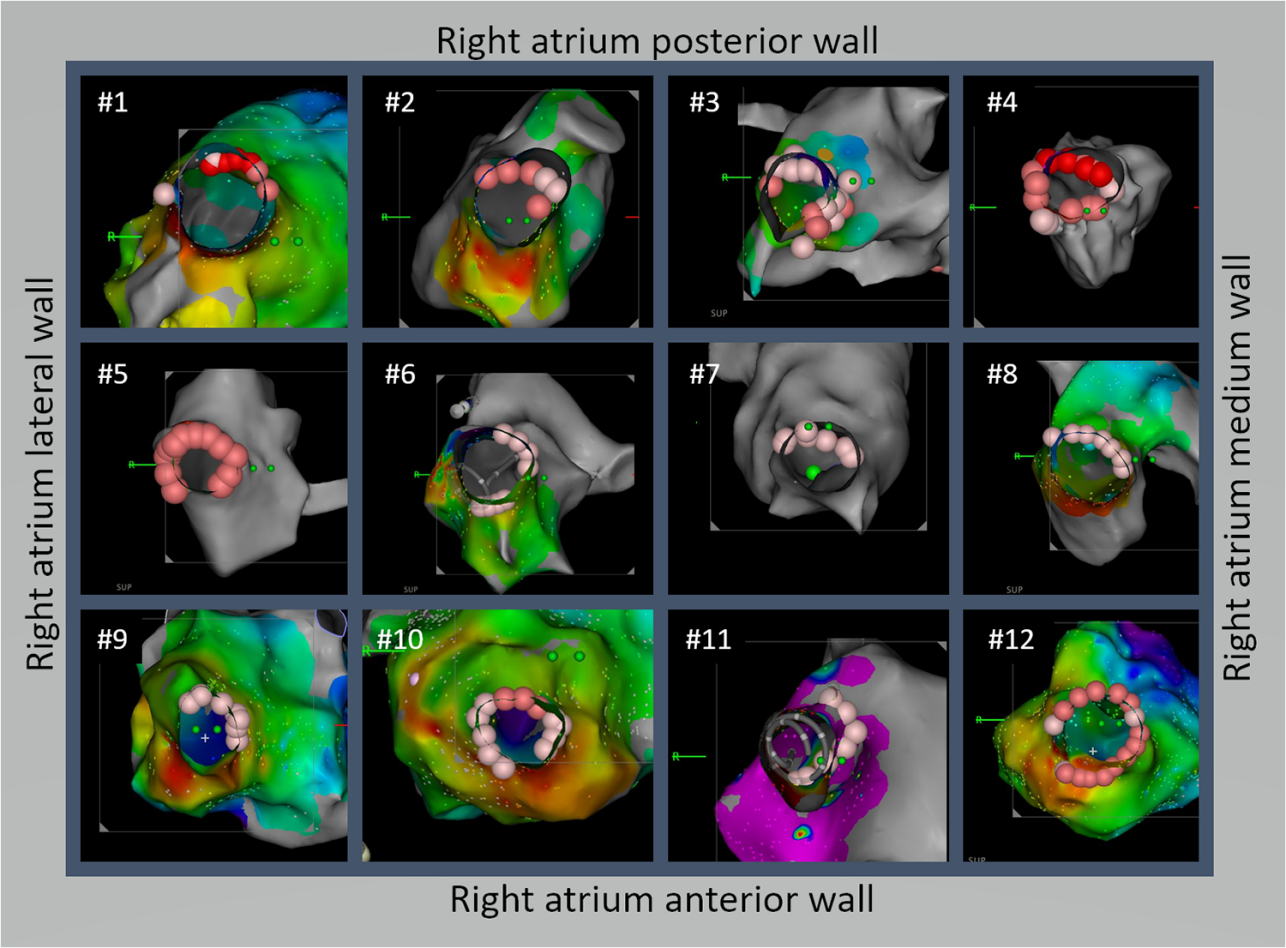
A novel technique for isolating the superior vena cava (SVC) using a non-circumferential approach from the anterior-medial to posterior-lateral aspects was effective in isolating the SVC and reducing the risk of SVC stricture. Detailed ablation line schemes are demonstrated for all 12 cases. Except #4, all patients achieved SVC isolation without circumferential ablation. Among the 12 patients, the true lateral segment was ablated in 3 patients (#4, #5, and #10) and 5 patients had the posterolateral segment ablated (#2, #3, #7, #8, and #12).

For SVC isolation, it was always performed after PV isolation. The location of the sinus node area was identified by finding the earliest activation site on the 3D map (Figure 1Ac, near the red area on the activation map). A 20-pole circular catheter was then placed inside the SVC where the local signals were clear (Figure 1B, yellow square frame). An ablation line was created approximately 1 cm above the sinus node (Figure 1Ab) and below the 20-pole circular catheter (30 W; ablation index for blunting of the local signal plus 50, usually around 300–350). The isolation line was created by applying energy point-by-point from the anterior/medial segment in a counterclockwise direction in the superior view until the SVC signals disappeared (Figure 2). The deflection point between the SVC and the right atrium (RA) was defined as the anatomical SVC-RA junction (Figure 1Ad). The distances from the anatomical SVC-RA junction (Figure 1Ad) to the end of the muscle sleeves (Figure 1Aa), ablation line (Figure 1Ab), and earliest activation site during sinus rhythm (Figure 1Ac) were measured. Results are presented as mean ± standard deviation.

The Kolmogorov–Smirnov test was used to confirm the normality of all variables.

## 3. Results

From March 2021 to the end of 2022, SVC isolation using the methods described above was performed in 12 patients, whose baseline characteristics are shown in Table 1. SVC isolation with bidirectional block was achieved in all patients. The distribution of ablation points for all patients in the superior view is shown in Figure 2. Notably, only one patient needed circumferential ablation to achieve SVC isolation. The length of the muscle sleeves, as measured from the SVC-RA junction to the end of the local signal, was 33.9 ± 6.7 mm. The distance between the SVC-RA junction and the ablation line was 16.8 ± 7.1 mm, while the distance between the ablation line and the sinus node was 11.9 ± 2.4 mm.

**Table 1:** Patients’ baseline characteristics.

Characteristic	*N* = 12
Sex (male), *n* (%)	7 (58.3%)
Age, years	66.1 ± 7.99
Body mass index, kg/m^2^	24.5 ± 3.6
Atrial fibrillation type, *n* (%):	
Paroxysmal	8 (66.7%)
Persistent	4 (33.3%)
Atrial fibrillation burden by Holter monitoring, %	46.3 ± 35.6 %
Coronary artery disease, *n* (%)	0 (0%)
Hypertension, *n* (%)	5 (41.7%)
Diabetes mellitus, *n* (%)	2 (16.7%)
Obese, *n* (%)	1 (8.3%)
Cardiac implantable electronic device, *n* (%)	0 (0%)
Cerebral vascular accident, *n* (%)	1 (8.3%)
Congestive heart failure, *n* (%)	2 (16.7%)
Chronic kidney disease, *n* (%)	1 (8.3%)
End-stage renal disease, *n* (%)	1 (8.3%)
CHA_2_DS_2_-VASc score, *n* (%):	
0	1 (8.3%)
1	4 (33.3%)
2	2 (16.7%)
3	3 (25%)
4	2 (16.7%)
Pulmonary vein isolation success, %	100%
SVC isolation success, %	100%
Length of the muscle sleeves, mm	33.9 ± 6.7
Distance between ablation line and SVC-RA junction, mm	16.8 ± 7.1
Distance between ablation line and sinus node, mm	11.9 ± 2.4
Fluoroscopy time, minute	7.2 ± 5.5
LA dwelling time, minute	99.9 ± 22.8
SVC mapping and isolation time, minute	19.7 ± 6.8

SVC, superior vena cava; RA, right atrium; LA, left atrium.

**Figure 3:**
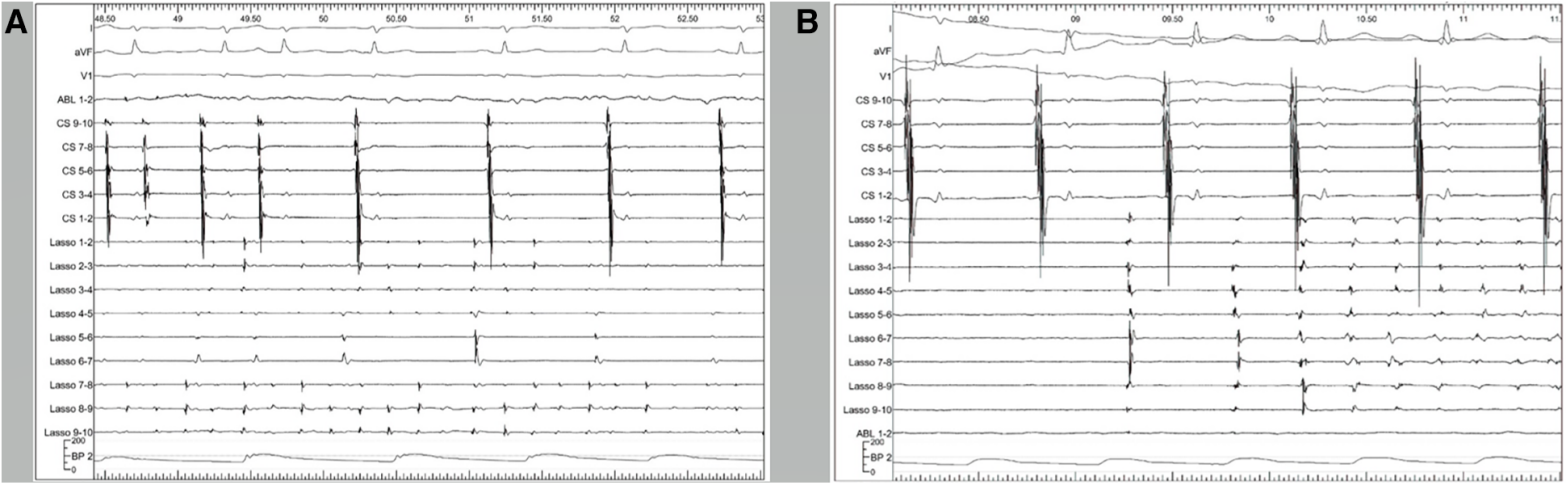
Representative polygraphic tracings from two of the patients. CS 9–10, 7–8, 5–6, 3–4, and 1–2 illustrate the electrograms of the CS from proximal to distal. The Lasso catheter was placed in the SVC and the tracings represent the local SVC electrograms. (**A**) At the start of the tracing, the atria (demonstrated by CS signals) is in fibrillation. Upon SVC isolation, the atria return to sinus rhythm while the SVC is still in fibrillation. After SVC isolation, the fibrillation inside the SVC remains. (**B**) Another patient with the same catheter placement. After SVC isolation, a 100-J electrical cardioversion was applied to convert the rhythm. Paroxysmal non-sustained atrial tachycardia was observed inside the SVC, which was dissociated from the right atrium and did not induce atrial fibrillation. CS, coronary sinus; SVC, superior vena cava.

As shown in Figure 1B, the activation map during sinus rhythm before ablation showed slow conduction at the anterolateral segments of the SVC. We achieved SVC isolation by ablating only the anterior-medial to posterior-lateral aspects of the SVC, sparing the anterolateral and lateral segments. In Figure 3, we present two cases of dissociated SVC signals (chaotic) from the RA (sinus rhythm) after creating a non-circumferential ablation line using our approach. During the follow-up period (11.6 ± 6.6 months), there were no clinical events of SVC stenosis, phrenic nerve injury, or sinus dysfunction.

## 4. Discussion

In this study, we present a novel approach for isolating the SVC that proved to be effective, with the potential to mitigate periprocedural risks. This approach involves starting the ablation from the anterior/medial segments and proceeding towards the posterior/lateral segments in a non-circumferential ablation line while applying a limited amount of energy. This approach may minimize the risks associated with SVC stricture and sinus node injury.

Although some patients show SVC firing, the indiscriminate isolation of the SVC in all patients with AF has not consistently yielded benefits. Chang et al. reported a cohort of 68 patients with AF originating from the SVC, showing acceptable AF-free outcomes following SVC isolation without PV isolation (4). Yoshiga further demonstrated the favorable effect of additional SVC isolation in patients with recurrent AF after previous PV isolation if no PV-reconnection was found (5). Overeinder et al. reported improved freedom from atrial tachycardias following SVC isolation with cryoballoon combined with PV isolation (6). However, a meta-analysis of randomized controlled trials revealed no significant benefit of empirical SVC isolation in addition to PV isolation (7, 8). Xu et al. reported a low prevalence (0.98%) of arrhythmogenic SVC in patients with long-standing persistent AF (8). These observations imply that arrhythmogenic SVC can be an AF trigger, but its prevalence might be too low for routine and empirical isolation. Higuchi et al. suggested that, in patients with long SVC sleeves (>30 mm) and high SVC potentials (>1.0 mV), the arrhythmogenic triggers of AF reside in the SVC (2). Therefore, SVC isolation might benefit only a few, carefully selected patients. In addition to SVC triggers, Palama et al. reported a significant proportion (19%) of supraventricular tachycardia inducibility in young patients referred for AF ablation, and ablation targeting only the supraventricular tachycardia prevented AF recurrence (9). In patients with AF, the triggers of AF should be carefully assessed in electrophysiological studies for adequate patient selection and improved outcomes.

Additionally, there are concerns regarding possible complications of SVC ablation, including SVC stenosis, sinus node injury, and phrenic nerve injury (10–12). To address these concerns, Gianni et al. proposed a segmental ablation approach that targets the septal region and sites of earliest activation in the posterior (electrically defined) SVC-RA junction and RA posterior wall, while avoiding ablation of the lateral segments where the phrenic nerve and sinus node are usually located (3). In their approach, a 20-pole catheter was positioned at the SVC-RA junction, and ablation was initiated at the septal and posterior aspects of the junction, continuing inferiorly to target the sites of early activation until electrical isolation was achieved. However, this strategy requires careful mapping to identify the earliest site on the posterior wall, which could make achieving SVC isolation more challenging. In our study, most patients (91.7%) achieved SVC isolation without the need for careful RA and SVC mapping. We further refined our approach by examining the activation map of the SVC and identifying the area of delayed conduction in the lateral wall. The ablation line was initiated from the anterior border of the region of slow conduction and additional ablation points were added in a counterclockwise direction from the superior view. Typically, the SVC signal disappears when the ablation line reaches the posterolateral region.

Previous studies differ in the definition of the SVC-RA junction. Gianni et al. and Higuchi et al. electrically defined the SVC-RA junction as the level at which sharp SVC potentials overlapped with blunt far-field atrial electrograms (2, 3). According to this definition, their SVC-RA junction is lower than where we placed the 20-pole circular catheter during ablation (Figure 1C, compared with Figure 1B), but we could not compare the precise position on the intracardiac echocardiography (ICE) image since our procedure did not use ICE. Additionally, we did not pay attention to the electrically defined SVC-RA junction because we designed the ablation line based on the sinus node location, with the primary concern of avoiding sinus node injury. Therefore, it is difficult to compare the distances obtained in this study with those reported by Gianni et al. However, both studies reported no reduction or absence of right diaphragmatic movement at the end of the procedure, suggesting that segmental ablation is feasible for SVC isolation.

In all 12 patients, natural conduction barriers were observed at the anterolateral aspect of the SVC. This phenomenon can be explained by embryogenesis. The SVC is formed by the merging of the right anterior and common cardinal veins during embryonic development. It then flows into the primitive RA through the right horn of the sinus venosus. As the sinus venosus is incorporated into the mature RA, it forms a smooth posterior wall known as the sinus venarum. The trabeculated anterolateral wall originating from the primitive RA is separated from the smooth posterior wall by the crista terminalis. The crista terminalis acts as an anisotropic conductor within the RA, which may explain why we were able to isolate the SVC using a non-circumferential ablation approach. Delayed or fragmented local signals were indications for SVC isolation in this study, which may have contributed to the high percentage of conduction barriers detected in the lateral SVC in our cohort. Higuchi et al. reported that SVC arrhythmogenic triggers of AF are associated with long SVC sleeves (>30 mm) and large SVC potentials (>1.0 mV) (2). However, the length of the SVC sleeves was measured fluoroscopically by quantitative angiography, in contrast to the current study, which measured SVC sleeve length on 3D maps. There may be an association between the length of the SVC sleeve and the presence of fragmented or delayed SVC potentials. Nonetheless, further studies are necessary to clarify this association and determine the optimal indicators for SVC isolation.

The limitations of this study are the small sample size and the lack of phrenic nerve function assessment and long term follow up results. A recent study showed that the typical course of the right phrenic nerve in the RA starts in the SVC at a lateral (50%), posterolateral (23%), or anterolateral (14%) position before descending either straight or with a slight curve towards the posterolateral wall of the RA (13). By initially delivering energy to the anterior segment, the lateral wall can be spared from ablation, reducing the chance of injury to the phrenic nerve (Figure 2). It is likely that ablation was performed close to the phrenic nerve in multiple patients in this case series. Although we did not map the course of the phrenic nerve before ablation, none of the patients experienced new-onset of dyspnea or chest discomfort, which are common manifestations of acute or chronic phrenic nerve palsy. We reviewed the medical records, and found that 6/12 patients had post-ablation chest x-ray during follow-up in the clinic for other reasons, and none had new-onset of diaphragm elevation. Since the incidence of phrenic nerve injury with SVC isolation is generally low (≤5%) and the case number of this series is small, the true risk of phrenic nerve injury with our C-shape approach may be underestimated. It would still be higher than that if performed by operators who carefully map the phrenic nerve prior to ablation and avoid it. Considering this, it is possible that our C-shaped ablation may reduce phrenic nerve injury if the phrenic nerve in a particular patient is located adjacent to the non-ablated segments. By starting ablation anteriorly and proceeding in counterclockwise fashion, bidirectional block can often be achieved without ablating the latera/posterolateral segments. However, as the phrenic nerve course is variable, mapping and avoiding it is still recommended prior to ablation. Moreover, the use of relatively low levels of energy for SVC isolation might have reduced the risk of phrenic nerve injury.

## 4. Conclusion

The proposed non-circumferential isolation line, located above the highest activation point of the sinus node and aiming to spare the lateral wall, is an effective approach to perform SVC isolation. This approach may reduce the risk of sinus node injury and latent SVC stenosis, but it warrants larger and long-term studies. It may be helpful in avoiding phrenic nerve injury in some patients, but care should be taken to map the course of phrenic nerve and avoid it. Our ablation strategy should be considered for patients who require SVC isolation.

## Data Availability

The original contributions presented in the study are included in the article, further inquiries can be directed to the corresponding author.
